# Carrying water may be a major contributor to disability from musculoskeletal disorders in low income countries: a cross-sectional survey in South Africa, Ghana and Vietnam

**DOI:** 10.7189/jogh.08.010406

**Published:** 2018-06

**Authors:** Jo-Anne Geere, Jamie Bartram, Laura Bates, Leslie Danquah, Barbara Evans, Michael B Fisher, Nora Groce, Batsirai Majuru, Michael M Mokoena, Murembiwa S Mukhola, Hung Nguyen-Viet, Phuc Pham Duc, Ashley Rhoderick Williams, Wolf-Peter Schmidt, Paul R Hunter

**Affiliations:** 1 Faculty of Medicine and Health Sciences, University of East Anglia, Norwich, United Kingdom; 2 The Water Institute, Gillings School of Global Public Health, University of North Carolina, Chapel Hill, USA; 3 Faculty of Public Health Engineering, University of Leeds, United Kingdom; 4 School of Geosciences, University of Energy and Natural Resources, Sunyani, Ghana; 5 Leonard Cheshire Disability & Inclusive Development Centre, Division of Epidemiology and Public Health, University College London, United Kingdom; 6 Department of Environmental Health, Tshwane University of Technology, South Africa; 7 Centre for Public Health and Ecosystem Research (CENPHER), Hanoi University of Public Health (HUPH), Hanoi, Vietnam; 8 International Livestock Research Institute (ILRI), Hanoi, Vietnam; 9 Environmental Health Group, Department for Disease Control, Faculty of Infectious and Tropical Diseases, London School of Hygiene and Tropical Medicine, United Kingdom

## Abstract

**Background:**

The Sustainable Development Goals include commitments to end poverty, and promote education for all, gender equality, the availability of water and decent work for all. An important constraint is the fact that each day, many millions of women and children, and much less frequently men, carry their household’s water home from off-plot sources. The burden of fetching water exacerbates gender inequality by keeping women out of education and paid employment. Despite speculation about the potential health impacts of fetching water, there is very little empirical evidence. We report the first large study of the health impacts of carrying water on women and children.

**Methods:**

A cross-sectional survey was conducted in South Africa, Ghana and Vietnam during 2012. It investigated water carrying methods and health status. Because areas of self-reported pain were correlated we undertook factor analysis of sites of reported pain, to interpret patterns of pain reporting. Regression analysis using Generalised Estimating Equations (GEE) investigated water carrying as a risk factor for general health and self-reported pain.

**Results:**

People who previously carried water had increased relative risk of reporting pain in the hands (risk ratio RR 3.62, 95% confidence interval CI 1.34 to 9.75) and upper back (RR 2.27, 95% CI 1.17 to 4.40), as did people who currently carry water (RR hand pain 3.11, 95% CI 1.34 to 7.23; RR upper back pain 2.16, 95% CI 1.25 to 3.73). The factor analysis results indicate that factor 1, ‘axial compression’, which is correlated with pain in the head and upper back, chest/ribs, hands, feet and abdomen/stomach, is associated with currently (0.30, 95% CI 0.17 to 0.43) or previously (0.21, 95% CI 0.01 to 0.42) carrying water. Factor 2, ‘soft tissue strain’, which is correlated with pain in the neck, shoulders/arms, lower back and hips/pelvis or legs, is marginally negatively associated with currently (-0.18, 95% CI -0.32 to -0.04) carrying water. The factor ‘axial compression’ was more strongly associated with carrying water containers on the head.

**Conclusions:**

Participants who reported a history of current or past water carrying more frequently reported pain in locations most likely to be associated with sustained spinal axial compression in the cervical region. Given the fact that cervical spinal conditions are globally one of the more common causes of disability, our findings suggest that water carrying, especially by head loading is a major contributing factor in musculoskeletal disease burden in low income countries. Our findings support the proposed indicator for monitoring SDG6.1: “Percentage of population using safely managed drinking water services at home.”

The United Nations Sustainable development goal (SDG) 6: ‘to ensure access to water and sanitation for all’ includes target 6.1: ‘By 2030, achieve universal and equitable access to safe and affordable drinking water for all’ [1]. The percentage of population using safely managed drinking water services *at home* has been proposed as the indicator for monitoring achievement of target 6.1 [2]. This represents a major shift toward recognising important differences in access, to distinguish water accessible within the home or yard, (‘at-house’ access), from water accessible at a supply point or source away from home (‘off-plot’ access). A difference is the work of water carriage required to bring water home from off-plot access. Perhaps the most influential study on the social and other impacts of water carriage was “Drawers of Water” [3], followed up some 30 years later by Drawers of Water II [4]. Conducted in East Africa, these studies raised awareness of the burden of fetching water for many Africans, especially women. The work of carrying water each day continues to mainly fall on women and girls, as reflected in a 2017 report of the WHO/UNICEF Joint Monitoring Programme, which found that women and girls were responsible for water collection in ‘eight out of ten households with water off premises’ with women responsible for water collection in 73.5% and girls in 6.9% of households of 61 DHS and MICs surveys [5]. Water carriage will be a major constraint on the achievement of diverse SDGs, including:

- SDG 1 “End poverty in all its forms” – when women have to spend much of their day fetching water they will not have the time to devote to activities that could increase their income.
- SDG 4 “Ensure inclusive and equitable quality education and promote lifelong learning opportunities for all” – when children, most often girls, spend time carrying water this prevents them from accessing education.
- SDG 5 “Achieve gender equality and empower all women and girls” – it is difficult to see how girls and women could be fully empowered when they spend much of their time fetching water [6].
- SDG 8 “Promote sustained, inclusive and sustainable economic growth, full and productive employment and decent work for all” – sustainable economic growth is less likely in those societies where half the work force spends much of its time fetching water.

Neither of the Drawers of Water studies were definitive about the impact of fetching water on health, or of health on capacity to fetch water. If carrying water adversely affects health, then it would also be a constraint on achieving SDG 3 “Ensure healthy lives and promote well-being for all at all ages”. Although there has been speculation, there has been little concrete evidence on the adverse health impacts of water carrying [7]. Research on water access has focussed on water source type, location or distance to water source rather than the work of water carriage, and on health outcomes such as acute diarrhoeal disease affecting children under 5 rather than household members who fetch water [8]. For example, in a systematic review Wang and Hunter [9] found an association between distances to water source and diarrhoeal disease. In another study Pickering and Davis found that both diarrhoeal disease and mortality in children under 5 were associated with time taken to fetch water from the nearest source [10]. However, in both these studies the adverse health impact was on children in the home and did not address the health of the person carrying the water.

Studies have reported detrimental effects of load carriage on the head [11-15] and limited evidence suggests that musculoskeletal disorders may be associated with water carrying [7,16-19]. Carrying water containers, particularly on the head (head loading), may impart physical stress to the bones and soft tissues of the neck and upper back through vertical compression or ‘axial loading’, and/or shear forces generated by translation in the horizontal plane [7,12,20,21]. The stress may tend to be greatest at specific regions or vertebral levels of the spine due to differences in structural anatomy of the vertebrae, with some variation due to age or gender [22], or an individual’s habits of posture and movement [23]. Peak or cumulative tissue stress loading during water carriage may be sufficient to produce pain, and if focussed at different regions of the spine may produce symptoms perceived in different locations of the body through well reported mechanisms of “referred” pain [7,23-26]. Therefore, given the substantial disease burden of musculoskeletal disorders in low and middle income countries [27,28], the substantial amount of women’s time spent carrying water [6,10] and the small amount of evidence suggesting an association between water carriage and musculoskeletal disorders [7,16-18], it is important to investigate and better understand how water carriage affects health, especially women’s and children’s health. Because a key feature of musculoskeletal disorders is pain, we hypothesized that water carriage would be significantly associated with self-reported pain and general health. We report the first large scale study undertaken across three countries to attempt to identify adverse health impacts on people who collect and who have to carry their family’s water home. Our objective was to evaluate the relationship between water carriage from an off-plot water source and physical health status as indicated by self-reported general health, pain and disability.

## METHODS

### Study design and setting

A cross-sectional survey was conducted, with recruitment and data collection occurring during June to December 2012 in Ghana, South Africa and Vietnam [29]. We selected geographical districts which were typical of low-income regions with sub-optimal water supply, and known to have communities with a mix of households with at-house and off-plot supplies, as the sampling frame in each country. We used a computer generated random number sequence to randomly select communities from each district to be included in the survey. In Ghana our research was conducted in four communities near Kumasi in the Ashanti region. All four communities were located around a main road and could broadly be defined as urban or peri-urban. Water was supplied through a combination of private taps, public taps, private boreholes and purchase of “sachet” water. In Vietnam our research was conducted in the remote, rural and mountainous Lao Cai province. The communities in Lao Cai were generally small scattered rural hamlets and most households accessed water from several sources, including piped water supply to the home, private boreholes and wells and public springs. In South Africa we carried out fieldwork in three peri-urban communities in Vhembe District in the northern parts of Limpopo Province in South Africa. Two communities were located in the dry, flat area west of Makhado town. The water sources here were communal taps or private drilled wells with either a yard tap or in-house connection. The third community was located in the foothills of the Soutpansberg mountain range. Shared water sources in the area are protected springs and communal taps, while some households had yard-taps or in-house taps.

### Sampling strategy

Assuming a sample size of 1000 participants and using the approach outlined by Hsieh et al [30], based upon simple logistic and linear regression, we calculated that a Power of 90% would be obtained even with a relatively small proportion of subjects with the outcome of interest. In South Africa 210 households were enrolled, in Ghana 255 and in Vietnam 208 generating a total of 997 participants who were asked about the variables of interest (**Table 1**). Stratified random sampling from within strata based on source of drinking water was used to recruit an even number of households with at-house and off-plot water supplies. All household members usually resident in selected households were eligible study participants.

**Table 1:** Demographic characteristics

	Ghana	South Africa	Vietnam	Total Number
Population of study communities	5160	-	-	N/A
Number of households (HH) in study communities	-	2113	264	N/A
HH enrolled in survey N (%)	255 (37.9%)	210 (31.2%)	208 (30.9%)	673 (100%)
Number of participants enrolled in survey N (%)	1326 (39.4%)	1230 (36.5%)	809 (24.1%)	3365 (100%)
Adults and children responding to pain, disability, general health and history of water carriage questions (1 adult and 1 child from each household) N (%)	397 (39.8%)	333 (33.4%)	267 (26.8%)	997 (100%)
Female gender whole survey: N (%)	753 (57.6%)	639 (52.0%)	401 (49.7%)	1793 (53.6%)
Female gender participants responding to pain, disability, general health and history of water carriage questions: N (%)	334 (84.8%)	234 (70.3%)	221 (82.8%)	789 (79.4%)
Mean age (standard deviation): whole survey	22.2 (23.5)	27.7 (21.3)	29.8 (20.9)	25.9 (22.4)
Mean age (standard deviation): participants responding to pain, disability, general health and history of water carriage questions	25.5 (16.3)	31.6 (22.2)	33.5 (20.5)	29.7 (19.8)
Adult* respondents to pain, disability, general health and history of water carriage questions with at home water supply N (%)	97 (43.1%)	103 (51.0%)	142 (77.2%)	342 (56.0%)
Adult* respondents to pain, disability, general health and history of water carriage questions with off plot water supply N (%)	128 (47.6%)	99 (49.0%)	42 (22.8%)	269 (44.0%)
Child† respondents to pain, disability, general health and history of water carriage questions with at home water supply N (%)	76 (45.5%)	73 (55.7%)	25 (30.5%)	174 (45.8%)
Child† respondents to pain, disability, general health and history of water carriage questions with off plot water supply N (%)	91 (54.5%)	58 (44.3%)	57 (69.5%)	206 (54.2%)

*Adult identified at Q14 as main survey respondent for households with at-house supply or usual water carrier for households with off-plot supply.

†Child identified at Q15 for response to health, disability and pain questions.

### Variables

The household survey collected demographic information about all household members, and included a questionnaire which asked respondents about exposure variables; whether their main water supply was currently obtained from an at-house or off-plot supply point, whether they currently or had ever carried water and their usual method of water carriage. Health outcome variables included self-reported pain, general health, disability and functioning; questions about these variableswere addressed to a subset of participants. The questions were administered to one adult respondent (93% women) and one child (57% girls) from each household. In houses with off-plot water supply, the questions were addressed to an adult and child identified by participants as a person in the household who would normally collect water, in households with at-house supply an adult and a child who would be responsible for collecting water if it were necessary. If a child was not present, the adult was asked to respond on their behalf.

A verbal descriptor of pain severity indicated as “mild”, “moderate” or “severe”, and experienced in the previous seven days [31-33], followed by additional questions to gather information about pain location, frequency and duration were used. To indicate general health, respondents were asked ‘In general, how would you rate your health today?’ and could select their response from a five point rating scale (1 = Very good; 2 = Good; 3 = Moderate; 4 = Bad; 5 = Very Bad). The short set of questions on disability developed and recommended for use in national surveys by the Washington Group on Disability Statistics [34] were used. Respondents were asked to rate whether they had difficulty in doing the activities of seeing, breathing, hearing, walking or climbing steps, remembering or concentrating, self-care and communicating. The response options were “no difficulty”, “some difficulty”, “a lot of difficulty” or ‘cannot do it at all’. Questions on functioning used by Atijosan et al, shown to have excellent reliability and validity in developing country settings [35] were used to indicate impairment of functioning. Respondents were asked whether they had difficulty using their arms, legs, any other part of their body such as the back or neck and whether they have “fits” or “epilepsy”. Response options were ‘no’, ‘yes, lasted less than 1 month’ or ‘yes, lasted more than one month or is permanent’. Information was also gathered on the potential confounding factors of age and gender.

Prior to commencement of any fieldwork activities, the data collection tools and protocol were revised, refined and standardised at a project workshop attended by the principle investigator (PI), co-investigators and field work team leads for each country in June 2012. The questions were then separately piloted in all three project locations and fieldworkers trained to administer the survey by the team leads within each country.

We addressed potential sources of bias by using stratified random sampling of households to reduce selection bias, limiting information about pain severity to pain experienced in the previous 7 days to reduce recall bias, training field-workers in a standardised interview protocol and monitoring the quality of data collection during fieldwork to reduce interviewer bias, and surveyed households about exposure variables and outcome variables on separate days to minimise response bias.

### Data analysis

Summary descriptive statistics for each country compare self-reported pain, general health, and disability of people with at-house supply to those using off-plot water supplies. Categorisation into at-house or off-plot water supply did not distinguish between people who did or did not engage in water carriage. Therefore, personal history of carrying water (by any method, categorised as currently carrying water, previously carried water or no history of water carriage) was used as the predictor variable. Analyses for health outcomes of pain reported in the previous seven days, pain location and self-rating of general health “today” were done using Generalised Estimating Equations (GEE) adjusted for age and gender and accounting for clustering at the household and country level. Participants with missing data were excluded from the analyses. Where the outcome variable was binary we used negative binomial regression with a log link, and where the outcome variable was scalar we used linear regression models. Because reporting of pain location at different parts of the body was correlated, we undertook a factor analysis of the different pain location variables. Two factors extracted from the overall data correlation matrix which accounted for the largest proportion of the total variance in the data were identified. Published literature was used to develop a theoretical construct offering a plausible explanation of the observed correlations between variables in each factor and to name each factor [36]. GEE with linear regression was then repeated for each factor and adjusted for age and gender, as well as to evaluate the strength of association with water carriage by head loading compared to other methods.

### Ethical review

This study was approved by the Ethical Committee of the School of International Development at the University of East Anglia for work in South Africa, the University of Leeds Ethical Committee, for work in Vietnam and the University of North Carolina for work in Ghana. In South Africa ethical clearance was also granted by the Tshwane University of Technology central ethical clearance committee, and the research team were invited by local chiefs within the study area to present their proposed research. In Ghana ethical approval was obtained from both the District Director of Health Services for Atwima Nwabiagya and the Atwima Nwabiagya District Assembly. In Vietnam, ethical clearance was obtained by the ethical research board of the Hanoi School of Public Health. Participants were included only after they had given informed written voluntary consent if ≥18 years old or if they and their guardian had given informed voluntary consent if <18 years old.

## RESULTS

### 

#### Patterns of water carriage

**Figure 1:**
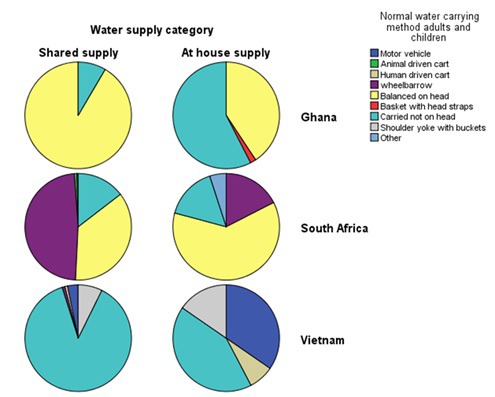
Water carriage method by supply type and country.

Respondents with at-house or off-plot water supplies were recruited in each country (**Table 1**). In South Africa and Ghana, substantial numbers of adults (South Africa SA 36.9%; Ghana GH 61.9%) and children (SA 19.2%; GH 43.4%) with at-house supply who were asked questions about general health, pain and disability, categorised themselves as currently carrying water. Whilst proportionately more women and children with off-plot supply in Ghana carried water by head loading, a considerable proportion of women and children with at- house supply also did so. A larger proportion of people with at-house supply in South Africa carried water by head loading compared to those with off-plot supply, as 42.4% of respondents with off-plot supply used a wheelbarrow to transport water (**Figure 1**). In both countries, participants reported episodes of interruption to at-house water supplies requiring water carriage from off-plot sources, which has also been reported in previous literature [37,38]. In all countries, substantial numbers of women with at-house supply had previously carried water (SA = 56.3%; GH 21.6%; Vietnam V 26.8%). The mean number of years in which they had engaged with water carrying were 25.4 (SD = 19.4) for South Africa, 19.7 (SD = 14.5) for Ghana and 7.1 (SD = 10.2) for Vietnam (Table S11 in **Online Supplementary Document(Online Supplementary Document)**).

#### Pain

Overall, comparing people with at-house vs off-plot supply within countries, there was no significant difference in reporting of pain experienced in the previous seven days (Table S1 and S4 in **Online Supplementary Document(Online Supplementary Document)**). Irrespective of at-house or off-plot supply categorisation, in South Africa proportionately fewer adults and children reported feeling pain in the previous seven days (SA adults 36.1%; children 4.6%) than in Ghana (adults 57.3%; children 18%) or Vietnam (adults 54.3%; children 21.7%).

**Figure 2:**
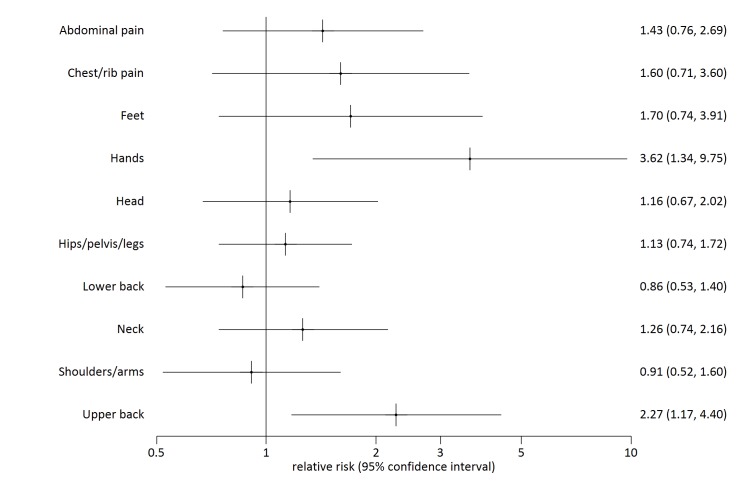
Sites of reported pain by past vs never water carrying.

**Figure 3:**
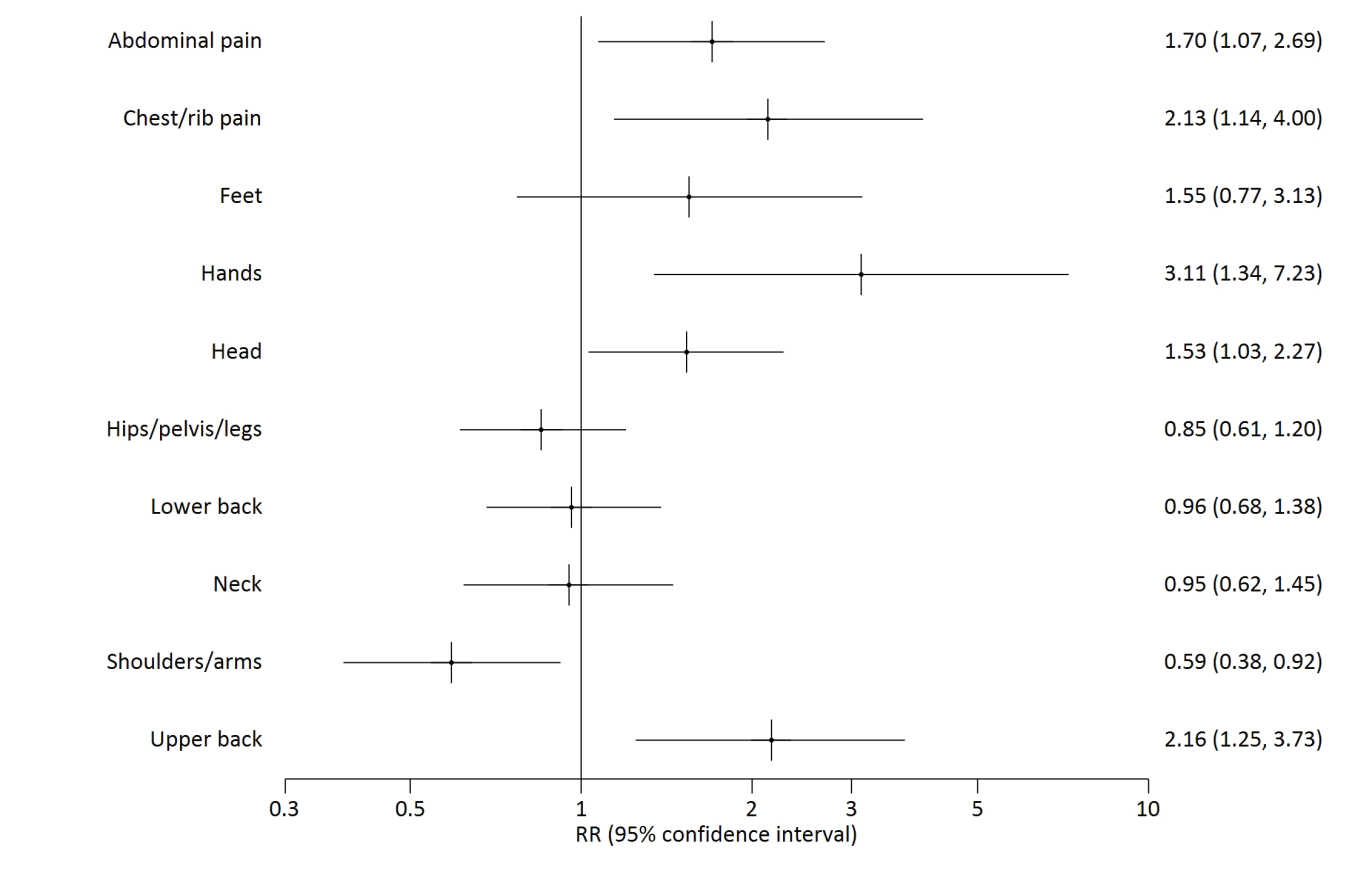
Sites of reported pain by current vs never water carrying.

History of water carriage did not significantly affect likelihood of reporting pain experienced in the previous seven days (**Table 2**). However pain reported in particular locations of the body was related to personal history of water carriage. Compared to people who had never carried water, people who previously carried water had increased relative risk of reporting pain in the hands and upper back (**Figure 2**, **Table 3**), as did people who currently carry water (**Figure 3**, **Table 3**). The factor analysis [36] results (**Table 4**) indicated that factor 1, interpreted as representing the effects of ‘axial compression’ is correlated with pain in the head and upper back, chest/ribs, hands, feet and abdomen/stomach, and is associated with currently (0.30, 95% CI 0.17 to 0.43) or previously (0.21, 95% CI 0.01 to 0.42) carrying water (**Table 5**). Factor 2, interpreted as indicating ‘soft tissue strain’ is correlated with pain in the neck, shoulders/arms, lower back and hips/pelvis or legs and is marginally negatively associated with currently carrying water (-0.18, 95% CI -0.32 to -0.04) (**Table 5**). Further analysis of people currently carrying water showed that the factor axial compression is significantly increased in people reporting head loading compared to those carrying by other means (**Table 5**). In an ordinal logistic regression analysis, those with higher axial compression scores tended to report shorter pain duration. Soft tissue strain scores were not associated with change in pain duration (**Table 6**).

**Table 2:** Adults and children self-report of pain in previous 7 days against history of water carriage

Pain previous 7 days	Predictor variable	N	RR	L95% CI	U95% CI	*P-*value
Adults	No history of water carriage	130	1			0.962
	Previous history of water carriage	145	0.97	0.77	1.23	
	Currently carries water	329	1.00	0.82	1.23	
Children	No history of water carriage	228	1			0.640
	Previous history of water carriage	11	NA			
	Currently carries water	139	0.89	0.55	1.44	

RR – relative risk, L95% CI – lower 95% confidence interval, U95% CI – upper95% confidence interval

**Table 3:** Relative risk of pain location from personal history of water carriage

Pain location	Predictor variable	N	RR	L95% CI	U95% CI	*P*-value
Abdominal pain	No history of water carriage	364	1			0.082
	Previous history of water carriage	159	1.43	0.76	2.69	
	Currently carries water	474	1.70	1.07	2.69	
Chest/rib pain	No history of water carriage	364	1			0.054
	Previous history of water carriage	159	1.60	0.71	3.60	
	Currently carries water	474	2.13	1.14	4.00	
Feet	No history of water carriage	364	1			0.394
	Previous history of water carriage	159	1.70	0.74	3.91	
	Currently carries water	474	1.55	0.77	3.13	
Hands	No history of water carriage	364	1			0.020
	Previous history of water carriage	159	3.62	1.34	9.75	
	Currently carries water	474	3.11	1.34	7.23	
Head	No history of water carriage	364	1			0.071
	Previous history of water carriage	159	1.16	0.67	2.02	
	Currently carries water	474	1.53	1.03	2.27	
Hips/pelvis/legs	No history of water carriage	364	1			0.373
	Previous history of water carriage	159	1.13	0.74	1.72	
	Currently carries water	474	0.85	0.61	1.20	
Lower back	No history of water carriage	364	1			0.828
	Previous history of water carriage	159	0.86	0.53	1.40	
	Currently carries water	474	0.96	0.68	1.38	
Neck	No history of water carriage	364	1			0.512
	Previous history of water carriage	159	1.26	0.74	2.16	
	Currently carries water	474	0.95	0.62	1.45	
Shoulders/arms	No history of water carriage	364	1			0.053
	Previous history of water carriage	159	0.91	0.52	1.60	
	Currently carries water	474	0.59	0.38	0.92	
Upper back	No history of water carriage	364	1			0.017
	Previous history of water carriage	159	2.27	1.17	4.40	
	Currently carries water	474	2.16	1.25	3.73	

RR - relative risk, L95% CI – lower 95% confidence interval; U95% CI – upper 95% confidence interval

**Table 4:** Factor analysis of self-reported pain locations*

Pain location	Factor 1: axial compression (correlation)	Factor 2: soft tissue strain (correlation)
Abdomen/stomach	0.632	0.131
Chest/ribs	0.706	0.151
Feet	0.695	0.221
Hands	0.706	0.266
Head	0.616	0.272
Hips/pelvis or legs	0.179	0.757
Lower back	0.223	0.750
Neck	0.340	0.697
Shoulders/arms	0.238	0.790
Upper back	0.608	0.347

*Extraction Method: Principal components; rotation: Equamax. Variance explained: 54.8%.

**Table 5:** Linear regression analysis of personal history of water carriage on Factor 1 (axial compression) and Factor 2 (soft tissue strain)

Factor correlated pain locations	Predictor variable	N	B	L95%CI	U05%CI	*P*-value
Factor 1: Axial compression	No history of water carriage	364	0			0.000045
	Previous history of water carriage	159	0.21	0.01	0.42	
	Currently carries water	474	0.30	0.17	0.43	
	Currently carries water – no head loading	214	0			0.034
	Currently carries water – head loading*	260	0.36	0.03	0.70	
Factor 2: Soft tissue strain	No history of water carriage	364	0			0.023
	Previous history of water carriage	159	-0.03	-0.25	0.19	
	Currently carries water	474	-0.18	-0.32	-0.04	
	Currently carries water – no head loading	214	0			0.64
	Currently carries water – head loading^1^	260	-0.07	-0.35	0.22	

B – linear regression coefficient, L95% CI – lower 95% confidence interval; U95% CI – upper 95% confidence interval

*Subgroup “currently carries water – head loading” only contains participants from South Africa and Ghana, as no-one in Vietnam carried water by head loading.

**Table 6:** Ordinal logistic regression of pain duration against Factor 1 (axial compression) and Factor 2 (soft tissue strain) with pain defined in three categories as <1 months, ≥1 months <3 months and ≥3 months (n = 333)

Predictor	OR	L95%CI	U95%CI	*P*-value
Factor 1: “Axial compression”	0.61	0.44	0.84	0.003
Factor 2: “Soft tissue strain”	1.30	0.59	2.86	0.521

OR – odds ratio, L95% CI – lower 95% confidence interval; U95% CI – upper 95% confidence interval

#### Physical functioning and disability

In Vietnam, proportionately more adults with at-house supply reported problems using their legs (ꭓ^2^ = 8.8; *P* = 0.01) or body (ꭓ^2^ = 8.8; *P* = 0.01) which had lasted for more than a month or was permanent (Table S7 and S8 in **Online Supplementary Document(Online Supplementary Document)**). Numbers of people reporting disability were very small and there were no significant differences in disability related to walking or self-care comparing people with at-house to those with off-plot supply within or across countries (Table S9 in **Online Supplementary Document(Online Supplementary Document)**).

#### General health

Most people in South Africa and Ghana rated their health as very good, good or moderate with no significant difference according to whether they had at-house or off-plot water supply (Tables S9 and S10 in **Online Supplementary Document(Online Supplementary Document)**). In Vietnam, most adults rated their general health as moderate or bad (76.1%), none rated it as very good. A larger proportion of adults in Vietnam with off-plot supply rated their health as bad, and a smaller proportion as moderate, compared to those with at-house supply (χ^2^ = 9.8; *P* = 0.01) (Table S9 in **Online Supplementary Document(Online Supplementary Document)**).

Interestingly, adults who previously carried water had a mean general health rating score 0.58 less (ie, healthier) than adults who never carried water (β = -0.58, 95% CI -0.80 to -0.35, *P* < 0.001) and adults who currently carried water had a mean general health rating score 0.91 less (ie, healthier) than adults who had never carried water (β = -0.91, 95% CI -1.12 to -0.70, *P* < 0.001) (**Table 7**). Children who currently carry water had a better mean score rating for general health than children who had never carried water (β = -0.20, 95% CI -0.37 to -0.31, *P* = 0.003). Children who previously carried water had a worse mean score rating for general health (β = 0.39, 95% CI 0.02 to 0.75), however the number of children in this category was very small (n = 10).

**Table 7:** Impact of personal history of water carriage rating of general health

General Health	Predictor variable	N	Β	L95%CI	U95%CI	*P*-value
Rating of general health today (adults)	No history of water carriage	123	0			<0.000001
	Previous history of water carriage	143	-0.58	-0.80	-0.35	
	Currently carries water	325	-0.91	-1.12	-0.70	
Rating of general health today (children)	No history of water carriage	204	0			0.003
	Previous history of water carriage	10	0.39	0.02	0.75	
	Currently carries water	128	-0.20	-0.37	-0.31	

B - linear regression coefficient L95% CI – lower 95% confidence interval; U95% CI – upper 95% confidence interval

## DISCUSSION

Current and past history of water carriage was associated with location of self-reported pain and ratings of general health. Reported pain locations were correlated and factor analysis revealed that Factor 1, which has been interpreted as the effects of ‘axial compression’, was associated with current or past water carriage, whilst Factor 2, interpreted as the effects of ‘soft tissue strain’ was slightly negatively associated with current water carriage. The factor ‘axial compression’ was most strongly associated with water carriage by head loading. The findings highlight that the experience of pain needs to be qualified in some detail to discriminate between people with different exposures to water carriage and with potentially different underlying causal mechanisms for their pain. The risk of reporting pain anywhere in the body indicated by a yes/no response to the question ‘in the past week (7 days) have you had any physical pain?’ was not significantly associated with different water supply or history of water carriage, likely reflecting the fact that physical pain is a common phenomenon in the general population. However, among those who did report pain, risk of reporting pain in specific parts of the body was significantly associated with history of water carriage. This is consistent with approaches to the clinical assessment of pain, in which location of pain is used to inform a differential diagnosis [39], and clinical pain research, in which the importance of pain location and multiple sites of pain is recognised [40]. Researchers should ask people where they feel pain, in addition to whether or not they have had any pain.

There is biological plausibility in the increased relative risk of pain in specific locations of the body in people with a current or past history of water carriage, as well as the correlation of pain areas in each factor and the association of axial compression with pain duration, water carriage and head loading in particular. Sustained axial compressive loading through the cervical spine and upper back, as occurs with carrying water filled containers on the head, is a plausible mechanism by which intervertebral discs or vertebrae of the upper cervical spine and cervico-thoracic junction may be stressed. Pal and Routal [22] described weight transmission through the cervical and thoracic spine and found that the second cervical vertebra and the cervico-thoracic junction anatomy indicate that load transference between the columns of the spine occurs at these levels, increasing tissue stress to make them more susceptible to bending or buckling deformity. Pal and Routal [22] cite Taylor and Twomey [41] to highlight that pubescent females have more slender spines and may be most vulnerable to adverse effects. Adverse effects due to axial loading stress could occur gradually, leading to degenerative changes in the intervertebral disc and associated zygoapophyseal joints, known as cervical spondylosis [12,13,42,43], or during head loading cause acute tissue stress or deformation to stimulate pain sensitive structures [20]. Through recognised pain referral mechanisms, such loading stress could cause pain to be perceived in the head, upper back and chest region, or hands. The pain from cervical degenerative disc disease tends to be associated with headache and inter-scapular (upper back) pain and may also cause irritation of spinal neural tissues to produce symptoms such as pain in the hands [24-26,44]. Because the cervical spinal canal protects both the spinal cord and peripheral nerve roots descending to lower regions of the spine, cervical problems can potentially cause more widespread symptoms and neurological impact than problems in the lumbar spine. Particularly in Africa, regular head loading has been linked to cervical spondylosis [12,13,42,43,45,46] and very heavy cervical loading to severe trauma and death [14]. People with cervical spondylosis causing spinal canal stenosis have been shown to be more at risk of serious spinal cord injury and its severely disabling consequences after even minor, indirect trauma to the cervical spine [47-51].

Our study is the first to find an association between water carriage and a pattern of correlated pain locations, which we believe is most likely due to a specific spinal musculoskeletal disorder caused by axial loading. Musculoskeletal disorders are within the top ten causes of years lived with disability in developing countries. Combined with fractures and soft tissue injuries they accounted for 20.8% of global years lived with disability in 2013, which would be even greater if years lived with disability due to sequelae of cervical disorders such as neurological impairment and headache were added [28]. Because water carriage is a modifiable activity, our study highlights at-house water supply as an important potential mechanism to reduce the burden of years lived with disability due to serious musculoskeletal disorders affecting children and adults, particularly women due to the gendered role of water carriage, in developing countries. Our findings also indicate that where people must continue to access their water from off-plot sources, enabling them to use alternative water carriage methods rather than head loading is a good first step. This could involve provision of affordable equipment, such as wheelbarrows, or improving access pathways to facilitate their use [16,19].

The correlation of pain locations with factor 2 (soft tissue strain) are more typical of simple non-specific spinal pain which produces somatic referred pain in the upper and lower limbs respectively. It may be due to the effects of soft tissue strain, for example generated by shear or translation stress, which can be reduced through better postural muscle control and functioning [23]. Water carriage and regularly walking to an off-plot water source could develop and maintain a level of muscle function and endurance which is slightly protective of joint or soft tissue strain [52-54]. It is plausible that whilst some individuals may experience pain associated with detrimental effects of axial compression; others may in fact benefit from the protective effect of exercise and better muscle control minimising soft tissue strain. Differential effects may be influenced by differences in total work load; for some water carriage may represent a major fraction of activity, and for others a minor fraction. Alternatively, water carriage patterns may be affected by unreliable water supplies [38], which could force women to collect as much water as possible when it is available, rather than pacing their work to avoid fatigue or pain due to tissue overload. Thus individual, task and environmental differences may lead to real differences in the experience of pain and therefore ability to fetch sufficient quantities of water, exacerbating inequalities in water access between households [16,27].

In all countries, proportionately more adults with off-plot supply, as compared to adults with at-house supply complained of pain lasting for less than a month, which was significantly associated with axial compression pain location patterns. The most common clinical pattern of degenerative disc disease or cervical spondylosis is episodic exacerbation or ‘flare up’ of symptoms followed by periods of remission or stability [44]. Constant pain is more typically a feature of serious pathology such as fracture, infection or cancer [55,56].

The better ratings of general health in those who previously or currently carry water may indicate some health benefits linked to increased physical activity. It could also indicate a greater sense of well-being linked to positive social interactions associated with water carriage. Results from the latest South African census also support the finding that the majority of the population in South Africa rate themselves as being in good health [57]. However, this finding may indicate that healthier people tend to become the household water carriers. Not everyone in a household will be tasked with carrying water and generally, people with more severe disabilities or illness are less likely to carry water [58]. Alternatively, in South Africa, the concept of ‘good health’ has been linked to ability to perform water carriage [17], an example of how cultural groups may define ‘health’ in terms of capacity to perform activities or to participate in society [59]. Such cultural differences in how health is conceptualised may to some extent influence self-rating of general health amongst water carriers, and may also explain the greater proportion of adults rating their health as moderate or bad in Vietnam.

The findings of no difference in perceived health with on-plot or off-plot supplies in Ghana and SA, yet better health among water carriers past and present than non-carriers indicate that there is an association with the activity of water carriage, but not with the type of household water supply. This is likely due to the large number of people categorised as having at-house water supply, who actually had previously carried water, or still have to carry water because of interruptions to supply, as shown in **Figure 1**. Our finding that water carriage is associated with pain location, and more weakly associated with better rating of general health may seem contradictory. However, it is possible to perceive that one’s general health, as a broader indicator, is good, but at the same time experience pain related to specific activities. For example, trained athletes or people who engage with high levels of physical activity may perceive that they have good general health related to their level of physical fitness, but do commonly experience musculoskeletal pain related to the activities they participate in. Pain location or patterns of pain are frequently used to indicate the underlying pathology or type of disorder causing the pain.

### Limitations

We did not include data about load carriage of materials other than water to reduce the size of the questionnaire and subsequent respondent burden, and to keep the focus on water access which was the primary aim of the research. However we do acknowledge that head loading of other materials, such as firewood [43,60], could be a confounding factor. In the multivariable analysis, we accounted for clustering at country, village and household level and this would have adjusted for the potential confounding effect of other activities associated with differences in geographic location, such as collecting wood, likely to be more similar within sites but different between the sites.

Although a limitation of the study was the use of a main survey respondent to answer health questions for household members if they were not present, the main respondent was most often an adult female who would be likely to have more insight into the health and medical history of her family members and have personal experience of water carriage herself. Clinical assessment of participants by a trained health professional would allow more informed interpretation of underlying causes of self-reported pain. Cause and effect must be established from a range of evidence including that derived from studies involving clinical assessment, and longitudinal cohort studies, which may reveal more complicated relationships between physical, psychological and social factors associated with water fetching and health. Nevertheless, previous studies have reported good correlation across populations between subjective symptoms and underlying radiological findings, and even in affluent countries, a diagnosis would usually be based on reported symptoms without reliance on ancillary investigations [44,61]. However, our study provides important evidence in support of the hypothesis that water carriage is significantly associated with pain and general health. Further research investigating the relationship between water fetching and health, ideally to include clinical assessment of water carriers by trained health professionals, is warranted.

## CONCLUSIONS

We have shown that people reporting a past or current history of water carriage were much more likely to report pain in locations typically associated with cervical compression syndromes. Cervical compression is associated with far more serious sequelae than back pain and can lead to serious long term disability in later life. Given that in 2015, 663 million people still use unimproved drinking water sources [5] it is likely that the burden of musculoskeletal disease from water carriage is substantial. Our findings support the ambition of the SDG target 6.1: ‘universal and equitable access to safe and affordable drinking water for all’ and indicate that to achieve the target, individual differences in the health impacts of water carriage must be recognised and addressed. Where access to water is likely to remain off-plot, alternative methods to load carriage on the head should be supported. Our findings also support the proposed shift to monitoring the percentage of the population using safely managed drinking water services at home as a key indicator.

## References

[1] United Nations. Goal 6: Ensure access to water and sanitation for all. 2015. Available: http://www.un.org/sustainabledevelopment/water-and-sanitation/. Accessed: 22 September 2017.

[2] WHO. UNICEF. WASH POST-2015: proposed targets and indicators for drinking-water, sanitation and hygiene. Geneva: WHO Press; 2014.

[3] White GF, Bradley DB, White AU. Drawers of Water. Chicago: University of Chicago; 1972.

[4] Thompson J, Porras IT, Tumwine JK, Mujwahuzi MR, Katui-Katua M, Johnstone N, et al. Drawers of Water II: 30 Years of Change in Domestic Water Use and Environmental Health in East Africa: Summary. 2002. Available: http://pubs.iied.org/pdfs/9049IIED.pdf. Accessed: 22 September 2017.

[5] WHO. UNICEF. Safely managed drinking water - thematic report on drinking water 2017. Geneva: World Health Organisation (WHO) and the United Nations Children's Fund (UNICEF); 2017.

[6] Puri L. Gender perspectives on water and food security. 2012. Available: http://www.unwomen.org/en/news/stories/2012/8/gender-perspectives-on-water-and-food-security. Accessed: 22 September 2017.

[7] Geere JA, Hunter PR, Jagals P (2010). Domestic water carrying and its implications for health: a review and mixed methods pilot study in Limpopo Province, South Africa.. Environ Health.

[8] Overbo A, Williams AR, Evans B, Hunter PR, Bartram J (2016). On-plot Drinking Water Supplies and health. A systematic Review.. Int J Hyg Environ Health.

[9] Wang X, Hunter PR (2010). A systematic review and meta-analysis of the association between self-reported diarrhoeal disease and distance from home to water source. Am J Trop Med Hyg.

[10] Pickering AJ, Davis J (2012). Freshwater availability and water fetching distance affect child health in sub-Saharan Africa.. Environ Sci Technol.

[11] Porter G, Hampshire K, Abane A, Munthali A, Robson E, Mashiri M (2012). Child Porterage and Africa’s Transport Gap: Evidence from Ghana, Malawi and South Africa.. World Dev.

[12] Jäger HJ, Gordon-Harris L, Mehring UM, Goetz GF, Mathias KD (1997). Degenerative change in the cervical spine and load-carrying on the head.. Skeletal Radiol.

[13] Joosab M, Torode M, Rao PV (1994). Preliminary findings on the effect of load-carrying to the structural integrity of the cervical spine.. Surg Radiol Anat.

[14] Levy LF (1968). Porter’s neck.. BMJ.

[15] Porter G, Hampshire K, Dunn C, Hall R, Levesley M, Burton K (2013). Health impacts of pedestrian head-loading: A review of the evidence with particular reference to women and children in sub-Saharan Africa.. Soc Sci Med.

[16] Geere J. Health impacts of water carriage. In: Bartram J, Baum R, Coclanis PA, Gute DM, Kay D, McFayden S, et al., editors. Routledge Handbook of Water and Health. London and New York: Routledge; 2015.

[17] Geere JL, Mokoena MM, Jagals P, Poland F, Hartley S (2010). How do children perceive health to be affected by domestic water carrying? Qualitative findings from a mixed methods study in rural South Africa.. Child Care Health Dev.

[18] Hemson D (2007). The toughest of chores: policy and practice in children collecting water in South Africa.. Policy Futures Educ.

[19] Robson E, Porter G, Hampshire K, Munthali A (2013). Heavy loads: children’s burdens of water carrying in Malawi.. Waterlines.

[20] Panjabi MM, Cholewicki J, Nibu K, Grauer J, Babat LB, Dvorak J (1998). Critical load of the human cervical spine: an in vitro experimental study.. Clin Biomech (Bristol, Avon).

[21] Panjabi MM, Summers DJ, Pelker RR, Videman T, Friedlaender GE, Southwick WO (1986). Three-dimensional load-displacement curves due to forces on the cervical spine.. J Orthop Res.

[22] Pal GP, Routal RV (1986). A study of weight transmission through the cervical and upper thoracic regions of the vertebral column in man.. J Anat.

[23] Jull G, Sterling M, Falla F, Treleaven J, O’Leary S. Whiplash, headache and neck pain. Research -based directions for physical therapies. Edinburgh: Churchill Livingstone Elsevier; 2008.

[24] Schellhas KP, Smith MD, Gundry CR, Pollei SR (1996). Cervical discogenic pain. Prospective correlation of magnetic resonance imaging and discography in asymptomatic subjects and pain sufferers.. Spine.

[25] Schellhas KP, Garvey TA, Johnson BA, Rothbart PJ, Pollei SR (2000). Cervical diskography: Analysis of provoked responses at C2-C3, C3-C4, and C4-C5.. AJNR Am J Neuroradiol.

[26] Slipman CW, Plastaras C, Patel R, Isaac Z, Chow D, Garvan C (2005). Provocative cervical discography symptom mapping.. Spine J.

[27] Hoy D, Geere J, Davatchi F, Meggitt B, Barrero LH (2014). A time for action: Opportunities for preventing the growing burden and disability from musculoskeletal conditions in low- and middle-income countries.. Best Pract Res Clin Rheumatol.

[28] Vos T, Barber RM, Bell B, Bertozzi-Villa A, Biryukov S, Bolliger I (2015). Global, regional, and national incidence, prevalence, and years lived with disability for 301 acute and chronic diseases and injuries in 188 countries, 1990–2013: a systematic analysis for the Global Burden of Disease Study 2013.. Lancet.

[29] Evans B, Bartram J, Hunter PR, Rhoderick Williams A, Geere J, Majuru B, et al. Public Health and Social Benefits of at-house Water Supplies. Final Report. University of Leeds: 2013.

[30] Hsieh FY, Bloch DA, Larsen MD (1998). A simple method of sample size calculation for linear and logistic regression.. Stat Med.

[31] Anderson KO (2005). Role of cutpoints: why grade pain intensity?. Pain.

[32] Singer AJ, Kowalska A, Thode HC (2001). Ability of patients to accurately recall the severity of acute painful events.. Acad Emerg Med.

[33] Broderick JE, Stone AA, Calvanese P, Schwartz JE, Turk DC (2006). Recalled pain ratings: A complex and poorly defined task.. J Pain.

[34] Madans JH Loeb ME Altman BM Measuring disability and monitoring the UN Convention on the Rights of Persons with Disabilities: the work of the Washington Group on Disability Statistics. BMC Public Health 2011 11 Suppl 4:S4 10.1186/1471-2458-11-S4-S4 21624190 PMC3104217

[35] Atijosan O, Kuper H, Rischewski D, Simms V, Lavy C (2007). Musculoskeletal impairment survey in Rwanda: Design of survey tool, survey methodology, and results of the pilot study (a cross sectional survey).. BMC Musculoskelet Disord.

[36] Portney LG, Watkins MP. Foundations of Clinical Research. Applicaitons to Practice. 2nd ed. New Jersey: Prentice Hall Health; 2000.

[37] Arnold M, VanDerslice JA, Taylor B, Benson S, Allen S, Johnson M (2013). Drinking water quality and source reliability in rural Ashanti region, Ghana.. J Water Health.

[38] Majuru B, Jagals P, Hunter PR (2012). Assessing rural small community water supply in Limpopo, South Africa: Water service benchmarks and reliability.. Sci Total Environ.

[39] Magee DJ. Orthopedic Physical Assessment. 4th ed. Philadelphia: Saunders; 2002.

[40] Carnes D, Parsons S, Ashby D, Breen A, Foster NE, Pincus T (2007). Chronic musculoskeletal pain rarely presents in a single body site: results from a UK population study.. Rheumatology.

[41] Taylor JR, Twomey LT (1984). Sexual dimorphism in human vertebral body shape.. J Anat.

[42] Jumah KB, Nyame PK (1994). Relationship between load carrying on the head and cervical spondylosis in Ghanaians.. West Afr J Med.

[43] Echarri JJ, Forriol F (2002). Effect of axial load on the cervical spine: a study of Congolese woodbearers.. Int Orthop.

[44] Clark CR. Degenerative Conditions of the Cervical Spine: Differential Diagnosis and Nonoperative Management. In: Frymoyer JW, editor. The Adult Spine Principles and Practice 1. Philadelphia: Lippincott - Raven; 1997. p. 1323-47.

[45] Adeloye A (1999). Syndrome of cervical spondylosis in Blantyre, Malawi.. East Afr Med J.

[46] Belachew DA, Schaller BJ, Guta Z (2007). Cervical spondylosis: a literature review with attention to the African population.. Arch Med Sci.

[47] Yoo DS, Lee S, Huh P, Kang S, Cho K (2010). Spinal cord injury in cervical spinal stenosis by minor trauma.. World Neurosurg.

[48] Fujiyoshi T, Yamazaki M, Okawa A, Kawabe J, Hayashi K, Endo T (2010). Static versus dynamic factors for the development of myelopathy in patients with cervical ossiﬁcation of the posterior longitudinal ligament.. J Clin Neurosci.

[49] Fengbin Y, Deyu C, Xinwei WYC, Jinhao M, Xinyuan L, Xiaowei L (2013). Trauma-induced spinal cord injury in cervical spondylotic myelopathy with or without lower cervical instability.. J Clin Neurosci.

[50] Regenbogen VS, Rogers LF (1986). AtIas SW, Kim KS. Cervical Spinal cord injuries in patients with cervical spondylosis.. AJR Am J Roentgenol.

[51] Ackland HM, Cameron PA, Varma DK, Fitt GJ, Cooper DJ, Wolfe R (2011). Cervical spine magnetic resonance imaging in alert, neurologically intact trauma patients with persistent midline tenderness and negative computed tomography results.. Ann Emerg Med.

[52] Heneweer H, Staes F, Aufdemkampe G, van Rijn M, Vanhees L (2011). Physical activity and low back pain: a systematic review of recent literature.. Eur Spine J.

[53] Heneweer H, Picavet SJ, Staes F, Kiers H, Vanhees L (2012). Physical fitness, rather than self-reported physical activities, is more strongly associated with low back pain: evidence from a working population.. Eur Spine J.

[54] Carroll LJ, Hogg-Johnson S, Côté P, van der Velde G, Holm LW, Carragee EJ (2009). Course and prognostic factors for neck pain in workers: Results of the Bone and Joint Decade 2000-2010 Task Force on neck pain and its associated disorders. J Manipulative Physiol Ther.

[55] Greene G (2001). Red Flags: essential factors in recognizing serious spinal pathology.. Man Ther.

[56] Guzman J, Haldeman S, Carroll LJ, Carragee EJ, Hurwitz EL, Peloso P (2008). Clinical practice implications of the Bone and Joint Decade 2000-2010 Task Force on neck pain and its associated disorders: from concepts and findings to recommendations.. Spine.

[57] Lehohla P. Census 2011 Methodology and highlights of key results. In: Africa SS, editor. Pretoria: Statistics South Africa; 2012.

[58] Groce N, Bailey N, Lang R, Trani JF, Kett M (2011). Water and sanitation issues for persons with disabilities in low- and middle-income countries: a literature review and discussion of implications for global health and international development.. J Water Health.

[59] Schatz E, Gilbert L (2014). “My legs affect me a lot. … i can no longer walk to the forest to fetch firewood”: challenges related to health and the performance of daily tasks for older women in a high HIV context.. Health Care Women Int.

[60] Lloyd R, Parr B, Davies S, Cooke C (2010). Subjective perceptions of load carriage on the head and back in Xhosa women.. Appl Ergon.

[61] Binder AI (2007). Cervical Spondylosis and Neck Pain.. BMJ.

