# Supplementary material for: Carrying water may be a major contributor to disability from musculoskeletal disorders in low income countries: a cross-sectional survey in South Africa, Ghana and Vietnam
Source: J Glob Health. 2018 Feb 16;8(1):010406. doi: 10.7189/jogh.08.010406 (PMC5825974; doi:10.7189/jogh.08.010406)
Supplement: Online Supplementary Document [file jogh-08-010406-s001.pdf]

Online Supplementary Document

Greere et al. Carrying water may be a major contributor to disability from musculoskeletal disorders in low income countries: a cross-sectional survey in South Africa, Ghana and Vietnam

J Glob Health 2018;8:010406

Table S1 Adults Self-reported pain and pain intensity (7 day recall; n 611)

Variable	Response category	GH n225 Frequency (%)		SA n202 Frequency (%)		V n184 Frequency (%)	
		At-house	Off-plot	At-house	Off-plot	At-house	Off-plot
Pain in past 7 days (Q24)	No	40 (41.2%)	56 (43.8%)	69 (67.0%)	60 (60.6%)	67 (47.2%)	17 (39.0%)
	Yes	57 (58.8%)	72 (56.3%)	34 (33.0%)	39 (39.4%)	75 (52.8%)	25 (58.5%)
	Total	97 (100.0%)	128 (100.0%)	103 (100.0%)	99 (100.0%)	142 (100.0%)	42 (97.6%)
Pain intensity (Q25)*	Mild	15 (15.5)	19 (14.8)	3 (2.9)	1 (1.0)	25 (17.6)	7 (16.7)
	Moderate	23 (23.7)	27 (21.1)	14 (13.6)	16 (16.2)	33 (23.2)	13 (31.0)
	Severe	19 (19.6)	26 (20.3)	17 (16.5)	22 (22.2)	17 (12.0)	5 (11.9)
	Total responses	57 (58.8)	72 (56.3)	34 (33.0)	39 (39.4)	75 (52.8)	25 (59.5)
	N/A	40 (41.2)	56 (43.8)	69 (67.0)	60 (60.6)	67 (47.2)	17 (40.5)

*Refers only to severity of pain experienced in last 7 days; N/A = not applicable, participant had not experienced pain in last 7 days

Table S2 Adults Self-reported pain descriptors (n 611)

Variable	Response category	GH n225 Frequency (%)		SA n202 Frequency (%)		V n184 Frequency (%)	
		At-house n97	Off-plot n128	At-house n103	Off-plot n99	At-house n142	Off-plot n42
Pain duration (Q26)	<1 month	32 (33.0)	52 (40.6)	16 (15.5)	23 (23.2)	12 (8.5)	8 (19.0)
	≥1 month <3 months	13 (13.4)	8 (6.3)	6 (5.8)	7 (7.1)	4 (2.8)	2 (4.8)
	≥3 months	10 (10.3)	12 (9.4)	9 (8.7)	8 (8.1)	58 (40.8)	15 (35.7)

	Total responses	55 (56.7)	72 (56.3)	31 (30.1)	38 (38.4)	74 (52.1)	25 (59.5)
	N/A	42 (43.3)	56 (43.8)	72 (69.9)	61 (61.6)	68 (47.9)	17 (40.5)
Constant pain (Q27)	always present	19 (19.6)	19 (14.8)	11 (10.7)	11 (11.1)	35 (24.6)	15 (35.7)
	comes and goes	38 (39.2)	53 (41.4)	23 (22.3)	27 (27.3)	40 (28.2)	10 (23.8)
	Total responses	57 (58.8)	72 (56.3)	34 (33.0)	38 (38.4)	75 (52.8)	25 (59.5)
	N/A	40 (41.2)	128 (100.0)	69 (67.0)	61 (61/6)	67 (47.2)	17 (40.5)

N/A = not applicable, participant had not experienced pain in last 7 days

Table S3 Adults Pain location

Variable	Response category	GH n225 Frequency (%)		SA n202 Frequency (%)		V n184 Frequency (%)	
		At-house n97	Off-plot n128	At-house n103	Off-plot n99	At-house n142	Off-plot n42
Pain location (Q28)*	Head	37 (38.1)	49 (38.3)	5 (4.9)	3 (3.0)	16 (11.3)	2 (4.8)
	Neck	21 (21.6)	28 (21.9)	2 (1.9)	2 (2.0)	35 (24.6)	5 (11.9)
	Shoulder/arms	14 (14.4)	21 (16.4)	3 (2.9)	3 (3.0)	35 (24.6)	7 (16.7)
	Hands	18 (18.6)	17 (13.3)	3 (2.9)	2 (2.0)	1 (0.7)	1 (2.4)
	Lower back	27 (27.8)	31 (24.2)	3 (2.9)	8 (8.1)	47 (33.1)	16 (38.1)
	Upper back	33 (34.0)	37 (28.9)	5 (4.9)	5 (5.1)	5 (3.5)	1 (2.4)
	Chest/ribs	19 (19.6)	26 (20.3)	3 (2.9)	8 (8.1)	3 (2.1)	2 (4.8)
	Abdomen/stomach	22 (22.7)	37 (28.9)	4 (3.9)	5 (5.1)	11 (7.7)	2 (4.8)
	Hips/pelvis or legs	25 (25.8)	34 (26.6)	7 (6.8)	9 (9.1)	44 (31.0)	17 (40.5)
	Feet	13 (13.4)	20 (15.6)	4 (3.9)	5 (5.1)	7 (4.9)	0 (0.0)

*Participants could indicate more than one body region as painful

Table S4 Children Self-reported pain and pain intensity (7 day recall; n380)

Variable	Response category	GH n167 Frequency (%)		SA n131 Frequency (%)		V n82 Frequency (%)	
		At-house	Off-plot	At-house	Off-plot	At-house	Off-plot
Pain in past 7 days (Q24)	No	58 (76.3)	79 (86.8)	70 (95.9)	55 (94.8)	46 (80.7)	19 (76.0)
	Yes	18 (23.7)	12 (13.2)	3 (4.1)	3 (5.2)	11 (19.3)	6 (24.0)
	Total	76 (100.0)	91 (100.0)	73 (100.0)	58 (100.0)	57 (100.0)	25 (100.0)

Pain intensity (Q25)*	Mild	4 (5.3)	4 (4.4)	0 (0.0)	0 (0.0)	7 (12.3)	3 (12.0)
	Moderate	7 (9.2)	4 (4.4)	1 (1.4)	3 (5.2)	4 (7.0)	1 (4.0)
	Severe	7 (9.2)	4 (4.4)	2 (2.7)	0 (0.0)	0 (0.0)	2 (8.0)
	Total responses	18 (23.7)	12 (13.2)	3 (4.1)	3 (5.2)	11 (19.3)	6 (24.0)
	N/A	58 (76.3)	79 (86.8)	70 (95.9)	55 (94.8)	46 (80.7)	19 (76.0)

*Refers only to severity of pain experienced in last 7 days; N/A = not applicable

Table S5 Children Self-reported pain descriptors (n 380)

Variable	Response category	GH n167 Frequency (%)		SA n131 Frequency (%)		V n82 Frequency (%)	
		At-house n76	Off-plot n91	At-house n73	Off-plot n58	At-house n25	Off-plot n57
Pain duration (Q26)	<1 month	15 (19.7)	10 (11.0)	3 (4.1)	2 (3.4)	6 (10.5)	3 (12.0)
	≥1 month <3 months	2 (2.6)	1(1.1)	0 (0.0)	1 (1.7)	0 (0.0)	2 (8.0)
	≥3 months	0 (0.0)	1 (1.1)	0 (0.0)	0 (0.0)	5 (8.8)	1 (4.0)
	Total responses	17 (22.4)	12 (13.2)	3 (4.1)	3 (5.2)	11 (19.3)	6 (24.0)
	Missing	59 (77.6)	79 (86.8)	70 (95.9)	55 (94.8)	46 (80.7)	19 (76.0)
Constant pain (Q27)	always present	3 (3.9)	4 (4.4)	0 (0.0)	0 (0.0)	1 (1.8)	2 (8.0)
	comes and goes	14 (18.4)	8 (8.8)	3 (4.1)	2 (3.4)	10 (17.5)	4 (16.0)
	Total responses	17 (22.4)	12 (13.2)	3 (4.1)	2 (3.4)	11 (19.3)	6 (24.0)
	Missing	59 (77.6)	79 (86.8)	70 (95.9)	56 (96.6)	46 (80.7)	19 (76.0)

N/A = not applicable, participant had not experienced pain in last 7 days

Table S6 Children Pain Location (n 380)

Variable	Response category	GH n167 Frequency (%)		SA n131 Frequency (%)		V n82 Frequency (%)	
		At-house n76	Off-plot n91	At-house n73	Off-plot n58	At-house n25	Off-plot n57
Pain location (Q28)*	Head	13 (17.1)	9 (9.9)	0 (0.0)	1 (1.7)	2 (3.5)	0 (0.0)
	Neck	5 (6.6)	1 (1.1)	0 (0.0)	0 (0.0)	1 (1.8)	0 (0.0)
	Shoulder/arms	2 (2.6)	0 (0.0)	0 (0.0)	0 (0.0)	0 (0.0)	0 (0.0)
	Hands	2 (2.6)	0 (0.0)	0 (0.0)	0 (0.0)	1 (1.8)	0 (0.0)

	Lower back	2 (2.6)	0 (0.0)	0 (0.0)	0 (0.0)	0 (0.0)	2 (8.0)
	Upper back	2 (2.6)	0 (0.0)	1 (1.4)	0 (0.0)	0 (0.0)	0 (0.0)
	Chest/ribs	3 (3.9)	1 (1.1)	1 (1.4)	0 (0.0)	1 (1.8)	1 (4.0)
	Abdomen/stomach	8 (10.5)	4 (4.4)	0 (0.0)	1 (1.7)	2 (3.5)	3 (12.0)
	Hips/pelvis or legs	3 (3.9)	1 (1.1)	0 (0.0)	0 (0.0)	4 (7.0)	0 (0.0)
	Feet	4 (5.3)	0 (0.0)	1 (1.4)	0 (0.0)	0 (0.0)	0 (0.0)

*Participants could indicate more than one body region as painful

Table S7 Self-reported physical functioning adults (n 611)

Variable	Response Category	GH n225 Frequency (%)		SA n202 Frequency (%)		V n184 Frequency (%)	
		At house	Off-plot	At house	Off-plot	At house	Off-plot
Difficulty using arms	No	65 (67.0)	87 (68.0)	86 (83.5)	79 (79.8)	101 (71.1)	36 (85.7)
	Yes (lasted < 1 month)	27 (27.8)	32 (25.0)	12 (11.7)	13 (13.1)	1 (0.7)	0 (0.0)
	Yes (lasted >1month/permanent)	5 (5.2)	7 (5.5)	4 (3.9)	5 (5.1)	40 (28.0)	6 (14.3)
	Total	97 (100.0)	126 (98.4)	102 (99.0)	97 (98.0)	142 (100.0)	42 (100.0)
Difficulty using legs	No	59 (60.8)	89 (69.5)	81 (78.6)	68 (68.7)	75 (52.8)	23 (54.8)
	Yes (lasted < 1 month)	28 (28.9)	31 (24.2)	12 (11.7)	18 (18.2)	2 (1.4)	5 (11.9)
	Yes (lasted >1month/permanent)	10 (10.3)	6 (4.7)	8 (7.8)	12 (12.1)	65 (45.8)	14 (33.3)
	Total	97 (100.0)	126 (98.4)	101 (98.1)	98 (99.0)	142 (100.0)	42 (100.0)
Difficulty using body: back/neck	No	51 (52.6)	72 (56.3)	92 (89.3)	82 (82.8)	66 (46.5)	24 (57.1)
	Yes (lasted < 1 month)	34 (35.1)	43 (33.6)	7 (6.8)	11 (11.1)	4 (2.8)	5 (11.9)
	Yes (lasted >1month/permanent)	11 (11.3)	11 (8.6)	1 (1.0)	5 (5.1)	71 (50.0)	13 (31.0)
	Total	96 (99.0)	126 (98.4)	100 (97.1)	98 (99.0)	141 (99.3)	42 (100.0)

Table S8 Self-reported physical functioning children (n 359)

Variable	Response Category	GH n156 Frequency (%)		SA n121 Frequency (%)		V n82 Frequency (%)	
		At house	Off-plot	At house	Off-plot	At house	Off-plot
Difficulty using arms	No	71 (93.4)	77 (84.6)	68 (93.2)	55 (94.8)	57 (100.0)	24 (96.0)
	Yes (lasted < 1 month)	4 (5.3)	9 (9.9)	1 (1.4)	0 (0.0)	0 (0.0)	0 (0.0)
	Yes (lasted >1month/permanent)	0 (0.0)	0 (0.0)	0 (0.0)	1 (1.7)	0 (0.0)	0 (0.0)
	Total	76 (100.0)	86 (94.5)	69 (94.5)	56 (96.6)	57 (100.0)	24 (96.0)

Difficulty using legs	No	66 (86.8)	81 (89.0)	68 (93.2)	55 (94.8)	53 (93.0)	25 (100.0)
	Yes (lasted < 1 month)	8 (10.5)	5 (5.5)	0 (0.0)	1 (1.7)	0 (0.0)	0 (0.0)
	Yes (lasted >1month/permanent)	2 (2.6)	0 (0.0)	0 (0.0)	0 (0.0)	4 (7.0)	0 (0.0)
	Total	76 (100.0)	86 (94.5)	68 (93.2)	56 (96.6)	57 (100.0)	25 (100.0)
Difficulty using body: back/neck	No	70 (92.1)	76 (83.5)	68 (93.2)	55 (94.8)	57 (100.0)	24 (96.0)
	Yes (lasted < 1 month)	6 (7.9)	9 (9.9)	0 (0.0)	1 (1.7)	0 (0.0)	0 (0.0)
	Yes (lasted >1month/permanent)	0 (0.0)	1 (1.1)	0 (0.0)	0 (0.0)	0 (0.0)	1 (4.0)
	Total	76 (100.0)	86 (94.5)	68 (93.2)	56 (96.6)	57 (100.0)	25 (100.0)

Table S9 General health and disability adults (n 611)

Variable	Response Category	GH n225 Frequency (%)		SA n202 Frequency (%)		V n184 Frequency (%)	
		At-house n97	Off-plot n128	At-house n103	Off-plot n99	At-house n142	Off-plot n42
Rating of general health	Very good	42 (43.3)	53 (41.4)	43 (41.7)	44 (44.4)	0 (0.0)	0 (0.0)
	Good	33 (34.0)	45 (35.2)	47 (45.6)	40 (40.4)	17 (12.0)	7 (16.7)
	Moderate	10 (10.3)	16 (12.5)	11 (10.7)	8 (8.1)	89 (62.7)	15 (35.7)
	Bad	9 (9.3)	12 (9.4)	2 (1.9)	6 (6.1)	23 (16.2)	13 (31.0)
	Very bad	2 (2.1)	1 (0.8)	0 (0.0)	1 (1.0)	1 (0.7)	1 (2.4)
	Total	96 (99.0)	127 (99.2)	103 (100)	99 (100.0)	130 (91.5)	36 (85.7)
Difficulty walking or climbing stairs	No difficulty	48 (49.5)	66 (51.6)	80 (77.7)	73 (73.7)	79 (55.6)	27 (64.3)
	Some difficulty	35 (36.1)	37 (28.9)	17 (16.5)	18 (18.2)	49 (34.5)	10 (23.8)
	A lot of difficulty	10 (10.3)	15 (11.7)	6 (5.8)	7 (7.1)	13 (9.2)	5 (11.9)
	Cannot do at all	1 (1.0)	0 (0.0)	0 (0.0)	1 (1.0)	1 (0.7)	0 (0.0)
	Total	94 (96.9)	118 (92.2)	103 (100.0)	99 (100.0)	142 (100.0)	42 (100.0)
Difficulty with self-care	No difficulty	76 (78.4)	105 (82.0)	99 (96.1)	96 (97.0)	109 (76.8)	36 (85.7)
	Some difficulty	15 (15.5)	11 (8.6)	2 (1.9)	1 (1.0)	31 (21.8)	6 (14.3)
	A lot of difficulty	3 (3.1)	3 (2.3)	2 (1.9)	1 (1.0)	2 (1.4)	0 (0.0)
	Cannot do at all	0 (0.0)	0 (0.0)	0 (0.0)	1 (1.0)	0 (0.0)	0 (0.0)
	Total	94 (96.9)	119 (93.0)	103 (100.0)	99 (100.0)	142 (100.0)	42 (100.0)

Table S10 General health and disability children (n 359)

Variable	Category	GH n156 Frequency (%)		SA n121 Frequency (%)		V n82 Frequency (%)	
		At-house n70	Off-plot n86	At-house n67	Off-plot n54	At-house n57	Off-plot n25
Rating of general health	Very good	49 (64.5)	61 (67.0)	39 (58.2)	27 (46.6)	0 (0.0)	1 (4.0)
	Good	20 (26.3)	20 (22.0)	20 (29.9)	21 (36.2)	21 (36.8)	7 (28.0)
	Moderate	3 (3.9)	3 (3.3)	4 (6.0)	1 (1.7)	14 (24.6)	5 (20.0)
	Bad	0 (0.0)	3 (3.3)	1 (1.5)	4 (6.9)	3 (5.3)	3 (12.0)
	Very bad	0 (0.0)	0 (0.0)	0 (0.0)	0 (0.0)	0 (0.0)	0 (0.0)
	Total	72 (94.7)	87 (95.6)	64 (95.5)	53 (91.4)	38 (66.7)	16 (64.0)
Difficulty walking or climbing stairs	No difficulty	75 (98.7)	90 (98.9)	73 (100.0)	56 (96.6)	57 (100.0)	25 (100.0)
	Some difficulty	1 (1.3)	1 (1.1)	0 (0.0)	1 (1.7)	0 (0.0)	0 (0.0)
	A lot of difficulty	0 (0.0)	0 (0.0)	0 (0.0)	0 (0.0)	0 (0.0)	0 (0.0)
	Cannot do at all	0 (0.0)	0 (0.0)	0 (0.0)	1 (1.7)	0 (0.0)	0 (0.0)
	Total	76 (100.0)	91 (100.0)	73 (100.0)	58 (100.0)	57 (100.0)	25 (100.0)
Difficulty with self-care	No difficulty	75 (98.7)	90 (98.9)	73 (100.0)	56 (96.6)	56 (98.2)	25 (100.0)
	Some difficulty	1 (1.3)	1 (1.1)	0 (0.0)	1 (1.7)	1 (1.8)	0 (0.0)
	A lot of difficulty	0 (0.0)	0 (0.0)	0 (0.0)	0 (0.0)	0 (0.0)	0 (0.0)
	Cannot do at all	0 (0.0)	0 (0.0)	0 (0.0)	1 (1.7)	0 (0.0)	0 (0.0)
	Total	76 (100.0)	91 (100.0)	73 (100.0)	58 (100.0)	57 (100.0)	25 (100.0)

TABLE S11 Past water carriers (Q56 ID) SA n = 207; GH n = 104; V n = 110

Variable	Country	N (%)	Mean (SD)	Median (Range)
Age when started carrying water	SA	144 (69.6%)	10.8 (8.4)	9 (5 – 65)
	GH	80 (76.9%)	12.5 (7.3)	10 (5 – 52)
	V	85 (%)	26.0 (11.5)	25 (5 – 54)
Age when stopped carrying water	SA	162 (78.3%)	37.2 (19.4)	33.5 (5 – 87)
	GH	82 (78.8%)	32.7 (12.6)	30 (14 – 70)
	V	101 (%)	34.5 (12.9)	34 (6 - 79)
Years water carrying	SA	130 (62.8%)	25.4 (19.4)	20 (0 – 81)
	GH	77 (74%)	19.7 (14.5)	18 (1 – 60)
	V	83 (%)	7.1 (10.2)	4 (0 – 66)

Figures comparing at-house versus shared supply in each country.

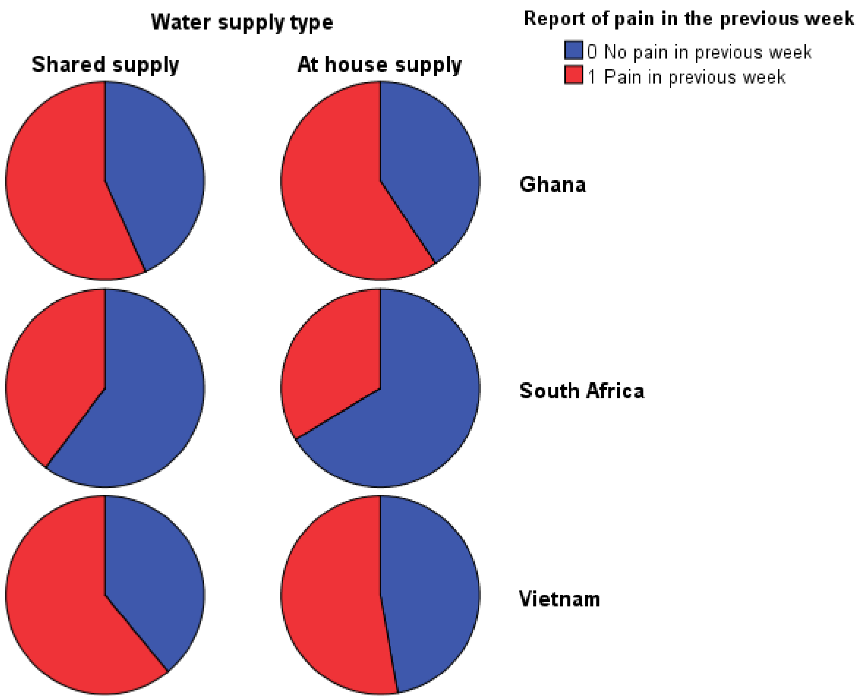

Figure 1: Report of pain in previous 7 days by water supply type and country

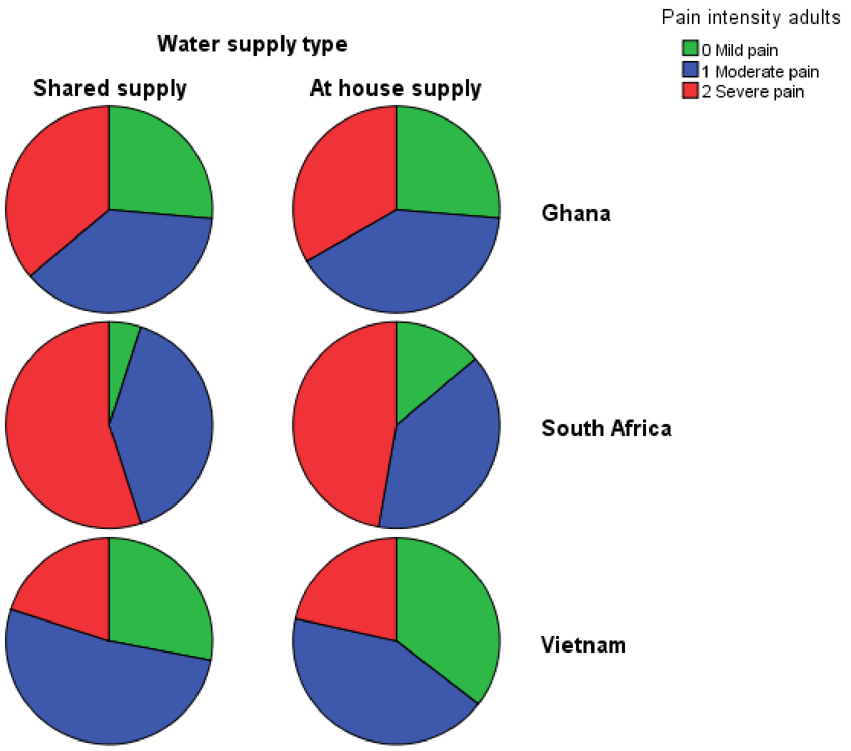

Figure 2: Reported pain intensity for adults according to water supply type and country

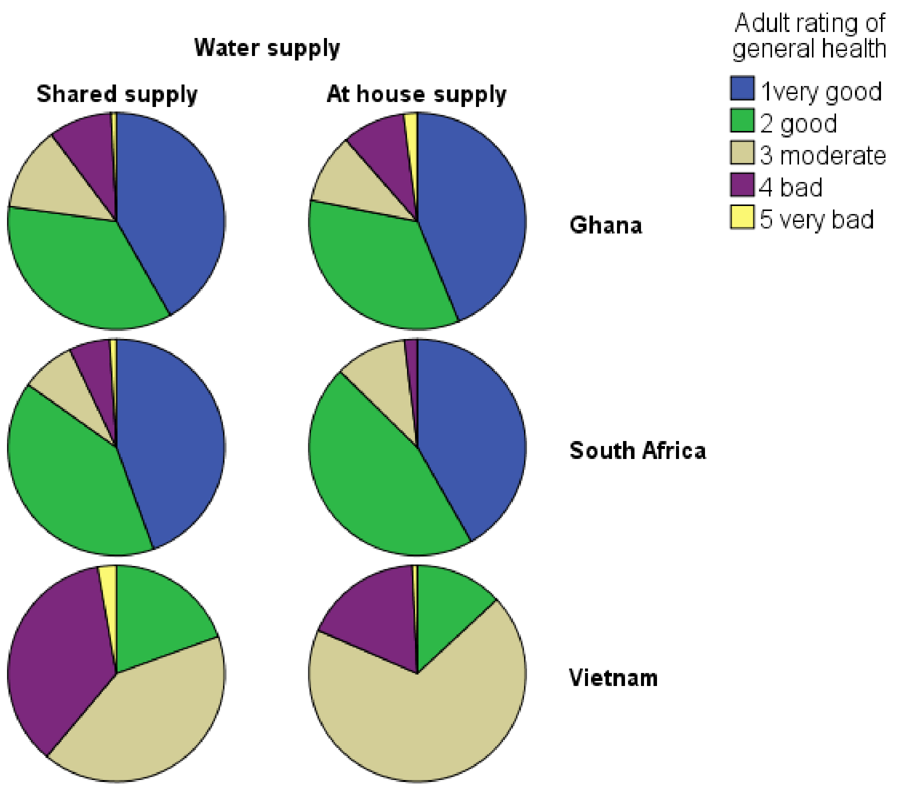

Figure 3: Comparison of adult rating of general health according to type of supply and country

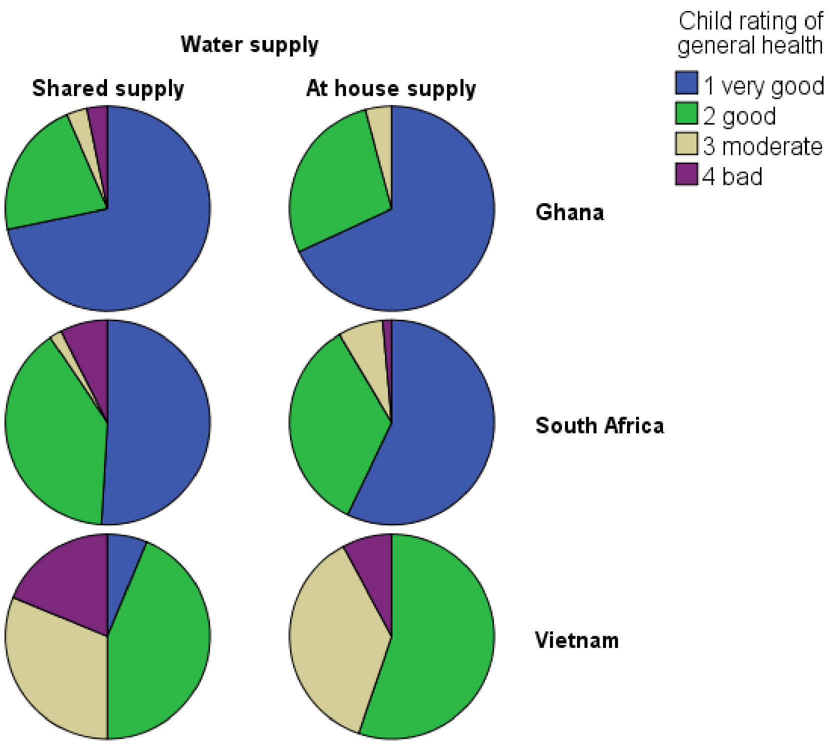

Figure 4: Comparison of child rating of general health according to type of supply and country

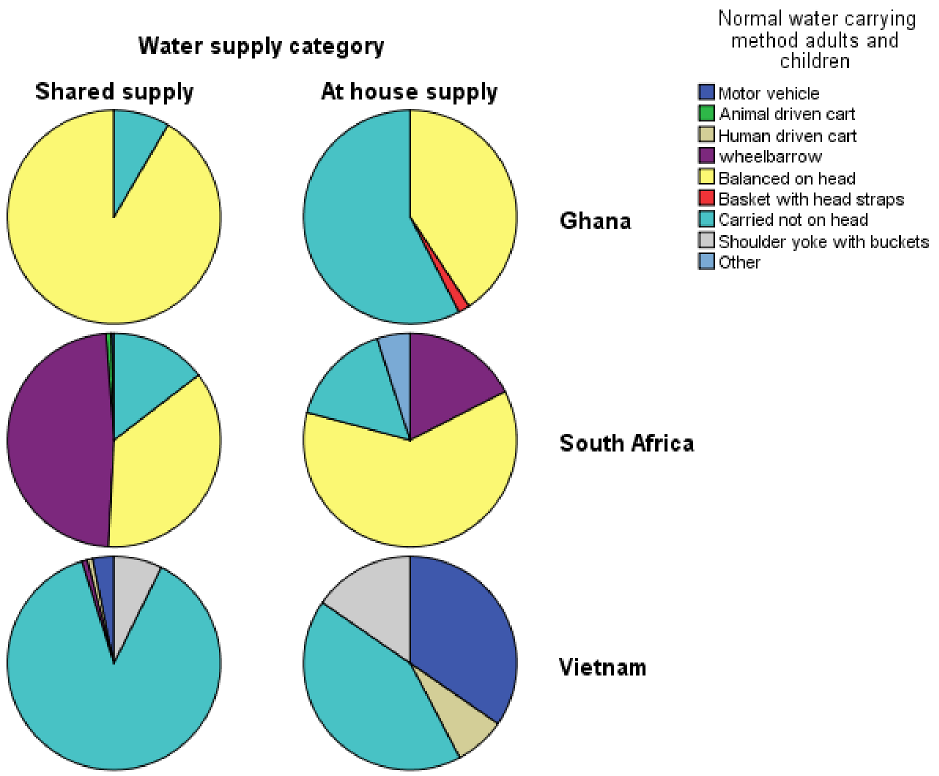

Figure 5: Water carrying method by supply type and country
